# Transcription Factor p73 Is a Predictor of Platinum Resistance and Promotes Aggressive Epithelial Ovarian Cancers

**DOI:** 10.3390/ijms26073239

**Published:** 2025-03-31

**Authors:** Ahmed Shoqafi, Reem Ali, Ayat Lashen, Jennie N. Jeyapalan, Asmaa Ibrahim, Michael S. Toss, Emad A. Rakha, Mashael Algethami, Shatha Alqahtani, Nigel P. Mongan, Dindial Ramotar, Srinivasan Madhusudan

**Affiliations:** 1 Naaz Coker Ovarian Cancer Research Centre, Biodiscovery Institute, School of Medicine, University of Nottingham, University Park, Nottingham NG7 3RD, UK; msxas29@exmail.nottingham.ac.uk (A.S.); mzxal1@exmail.nottingham.ac.uk (A.L.); plzjnj@exmail.nottingham.ac.uk (J.N.J.); mrzear1@exmail.nottingham.ac.uk (E.A.R.); msxma58@exmail.nottingham.ac.uk (M.A.); mzxsa20@exmail.nottingham.ac.uk (S.A.); svznpm@exmail.nottingham.ac.uk (N.P.M.); 2 Division of Biological and Biomedical Sciences, College of Health and Life Sciences, Hamad Bin Khalifa University, Education City, Qatar Foundation, Doha P.O. Box 34110, Qatar; reaali@hbku.edu.qa (R.A.); dramotar@hbku.edu.qa (D.R.); 3 Department of Pathology, Nottingham University Hospitals, City Hospital Campus, Nottingham NG5 1PB, UK; mzxai2@exmail.nottingham.ac.uk (A.I.); msamt9@exmail.nottingham.ac.uk (M.S.T.); 4 Centre for Cancer Sciences, University of Nottingham, Sutton Bonington Campus, Sutton Bonington, Leicestershire LE12 5RD, UK; 5 Department of Pharmacology, Weill Cornell Medicine, New York, NY 10065, USA; 6 Department of Oncology, Nottingham University Hospitals, City Hospital Campus, Nottingham NG5 1PB, UK

**Keywords:** ovarian cancers, platinum resistance, DNA repair, p73, p53, biomarker

## Abstract

Resistance to platinum-based chemotherapy is a major clinical problem in ovarian cancers. The development of predictive biomarkers and therapeutic approaches is an area of unmet need. p73, a member of the p53 family of transcription factors, has essential functions during DNA repair, proliferation, invasion, and apoptosis. The role of p73 in ovarian cancer pathogenesis and response to therapy is largely unknown. The clinicopathological significance of p73 protein expression was evaluated in 278 human ovarian cancers. *TP73* transcripts were investigated in publicly available clinical data sets (n = 522) and bioinformatics analysis was completed in the ovarian TCGA cohort (n = 182). Preclinically, p73 was overexpressed in A2780 platinum-sensitive ovarian cancer cells or depleted in platinum-resistant A2780cis cells and investigated for aggressive phenotypes, as well as platinum sensitivity. High p73 protein expression was linked with high grade (*p* < 0.001), advanced-stage disease (*p* = 0.002), and shorter progression-free survival (*p* < 0.0001). *TP73* transcripts were significantly higher in tumours compared to normal tissue (*p* < 0.0001) and linked with shorter PFS (*p* = 0.047). Preclinically, p73 overexpression in A2780 cells increased proliferation, invasion, spheroid formation, and DNA repair capacity, and was associated with the upregulation of multiple DNA repair and platinum resistance-associated genes. In contrast, p73 deletion in A2780cis led to reduced proliferation and enhanced sensitivity to cisplatin, along with DNA double-strand break accumulation, G2/M cell cycle arrest, and increased apoptosis. We conclude that p73 is a predictor of platinum resistance. p73 can be exploited for targeted ovarian cancer therapy.

## 1. Introduction

p73 belongs to the p53 family of transcription factors [1,2,3]. p73 is a key player during neurodevelopment, tissue homeostasis, and cancer [1,2,4,5,6,7,8,9]. p73 has three basic functional domains: the transactivation domain (TA), the core DNA-binding domain (DBD), and the oligomerization domain (OD). In addition, p73 has a SAM (sterile alpha motif) domain in the C-terminus that promotes protein stability [1,5,9]. p73 is a structural and functional homolog of p53. Unlike p53, p73 is rarely mutated in solid tumours, including epithelial ovarian cancers [1,2,4,5,6,7,8,9]. In contrast, mutations of *p53* have been described in about 50% of advanced ovarian cancers (FIGO stages III and IV) and in 15% of early-stage cancers (FIGO stages I and II) [10].

p73 is located on human chromosome 1p36, a region that is a recurrent, specific breakpoint site of translocation in ovarian cancers [1]. Several isoforms can be transcribed from the *TP73* gene locus [1,5,9]. ΔN (Delta N) p73 refers to a group of N-terminally truncated isoforms of p73. ΔNp73 refers to the ΔN isoform of p73, which retains exon 2 but lacks exon 1. This isoform is also N-terminally truncated and does not include the transactivation domain. The η* isoform is another alternative splice variant of p73 and less common compared to the ΔN and ΔNp73 isoforms. These isoforms generated by alternative splicing in the 5′end include TA, ΔN, ΔEx2p73, ΔEx2/3p73, and ΔN’p73 isoforms, or C-terminal splice variants such as α, β, γ, δ, ε, ζ, η, η∗, η1, and θ isoforms. The isoforms ΔEx2p73, ΔEx2/3p73, and ΔN’p73 partially or entirely lack the transactivation domain and can have a dominant negative (DN) effect over TA isoforms. The ΔNp73 isoforms, along with ΔN, constitute the so-called DN isoforms. Previous studies have shown that the TAp73 isoform is a tumour suppressor, but ΔNp73 is an oncogene [11]. While total p73 knockout mice show developmental abnormalities, p73+/− heterozygous mice are prone to develop cancers. Moreover, TAp73−/− mice also show an increased susceptibility to cancer, but ΔNp73−/− mice do not [1]. These studies suggest complex biological functions for various isoforms of p73 [1,5,9]. In fact, p73 isoforms can form diverse protein–protein interactions with many nuclear (such as MDM2, YAP1, CDK complex, WT1, Sp1, MCL1, SUMO1, PTEN, MM1, and others) and cytoplasmic proteins (such as NGFR, PKP1, KCK, NEDL2, amphiphysinIIb-1, Wwox, and others) to accomplish various biological functions (reviewed in [1,5,9]). Although complex, the overall biological effect of p73 isoforms is influenced by the TA/DN isoform ratio, as opposed to the overexpression of a specific p73 isoform or a specific class of p73 isoforms in cells [1,5,9].

In cancers, p73 is involved in genomic instability, pro-proliferative signalling, the evasion of growth suppression, the activation of invasion and metastasis, angiogenesis, immune evasion, altered cellular energetics, neo-neurogenesis, and response to cytotoxic therapy [1,2,4,5,6,7,8,9]. p73 dysregulation has been reported in solid tumours. In ovarian cancer cell lines, the downregulation of *TP73* transcripts by epigenetic silencing has been reported [12]. In human ovarian tumours, reports suggest higher levels of p73 in advanced ovarian cancer compared to early-stage disease [13,14]. However, in contrast to p53, the clinicopathological significance of p73 in ovarian cancer is largely unknown due to small sample sizes in previous studies. In the current study, we evaluate the clinical significance of p73 in a large clinical cohort of ovarian cancer in the context of wild-type or mutant p53. Pre-clinically, p73 was overexpressed and depleted, and platinum sensitivity was evaluated in ovarian cancer cell lines.

## 2. Results

### 2.1. p73 Protein Expression

**Figure 1:**
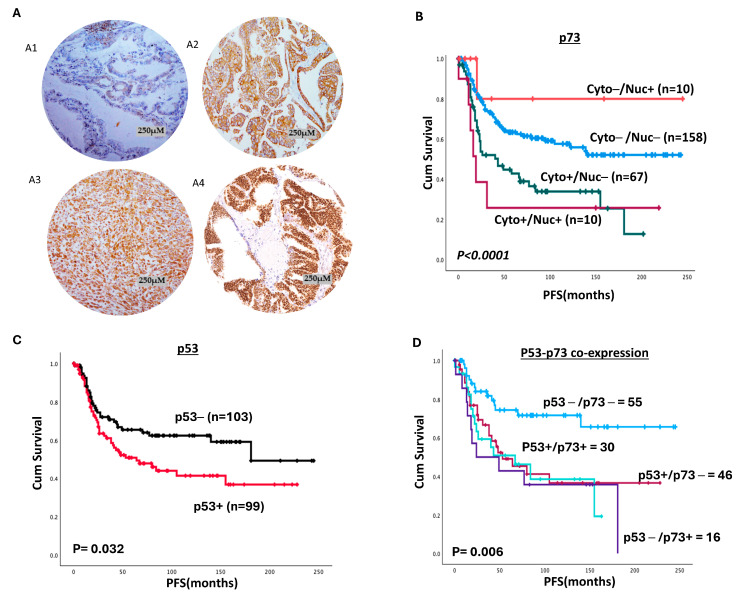
p73 and p53 protein expression in ovarian cancers. (**A1**) Photomicrographs showing p73-negative ovarian cancers. (**A2**) Photomicrographs showing cytoplasmic immunohistochemical staining of p73 in ovarian cancer tissue. (**A3**) Photomicrographs showing nuclear immunohistochemical staining of p73 in ovarian cancer tissue. (**A4**) Photomicrographs showing nuclear immunohistochemical staining of p53 in ovarian cancer tissue. (**B**) Kaplan–Meier curve for p73 nuclear/cytoplasmic co-expression and progression-free survival (PFS). (**C**) Kaplan–Meier curve for p53 nuclear protein expression and PFS. (**D**) Kaplan–Meier curve for p73/p53 co-expression and progression-free survival (PFS). Although 161 cases had expression data available for both p73 and p53 expression, clinical outcome data were available for only 147/161 patients and included in the survival analysis [light-blue line = p53−/p73−, aqua line = p53+/p73+, dark-red line = p53+/p73−, purple line = p53−/p73+]. SPSS software version 29 from IBM was used for Kaplan–Meier survival curves.

A total of 278 tumours were evaluable for p73 expression. We observed nuclear and cytoplasmic staining for p73 (Figure 1(A1–A3)). High nuclear p73 expression was seen in 22/278 (8%), and high cytoplasmic p73 expression was observed in 89/278 (32%). The remaining samples were not assessed due to missing cores after IHC or an insufficient number of tumour cells for scoring. Low nuclear p73 was significantly associated with serous tumours (*p* = 0.028) (Supplementary Table S3). On the other hand, high cytoplasmic p73 was linked with serous tumours (*p* < 0.0001), high grade (*p* = 0.006), and high pathology stage (*p* = 0.025) (Supplementary Table S3). No residual tumour after surgery was more likely in low-cytoplasmic p73 tumours (*p* < 0.0001) (Supplementary Table S4). We then performed co-expression analysis (Table 1) and observed that tumours with high-cytoplasmic/low-nuclear p73 were associated with serous tumours (*p* < 0.0001). No residual disease after surgery was more likely in tumours with low-cytoplasmic/low-nuclear p73 expression (*p* = 0.003).

High cytoplasmic p73 was significantly linked with poor progression-free survival (PFS) (*p* < 0.0001) (Supplementary Figure S1A), but not overall survival (OS) (*p* = 0.088) (Supplementary Figure S1B). Nuclear p73 was not associated with PFS or OS (*p* = 0.731 and *p* = 0.219 respectively) (Supplementary Figure S1C,D). We then performed co-expression analysis and observed that high-cytoplasmic/high-nuclear p73 tumours had poor PFS (*p* < 0.0001) (Figure 1B) but not OS (*p* = 0.166) (Supplementary Figure S1E).

### 2.2. p73/p53 Co-Expression

p53 was analysed in 224 cases due to missing cores after IHC or an insufficient number of tumour cells for scoring. Nuclear p53 staining was observed in 111/224 (49.5%) tumours (Figure 1(A4)) and was significantly associated with serous-type tumours (*p* = 0.001), high-grade cancers (*p* = 0.001) (Supplementary Table S5), and poor PFS (*p* = 0.032) (Figure 1C), but not OS (*p* = 0.462) (Supplementary Figure S1F).

We then conducted p73/p53 co-expression analysis (Supplementary Table S6). For analyses involving both markers, 161 cases had expression data available for both p73 and p53 expression. The missed cores were different between p73 and p53, as each marker was stained at different occasions (both low in 60 cases, both high in 34 cases, high p53 with low p73 in 50 cases, and high p73 with low p53 in 17 cases). Additionally, since outcome data were unavailable for some patients, the number of cases included in the survival analysis was lower than in the clinicopathological analysis.

High-p73/high-p53 tumours were likely to be serous tumours (*p* = 0.011), whereas low-p73/low-p53 staining was more likely in low-grade tumours (*p* = 0.046) with no residual tumours after surgery (*p* = 0.005) (Supplementary Table S6), showing better PFS (*p* = 0.006) (Figure 1D) but not OS (*p* = 0.332) (Supplementary Figure S2A). Taken together, these data provide evidence that p73 protein expression is associated with poor PFS and could be a predictor of platinum resistance. We proceeded to evaluate *p73* transcripts in publicly available datasets.

### 2.3. p73 Transcripts

**Figure 2:**
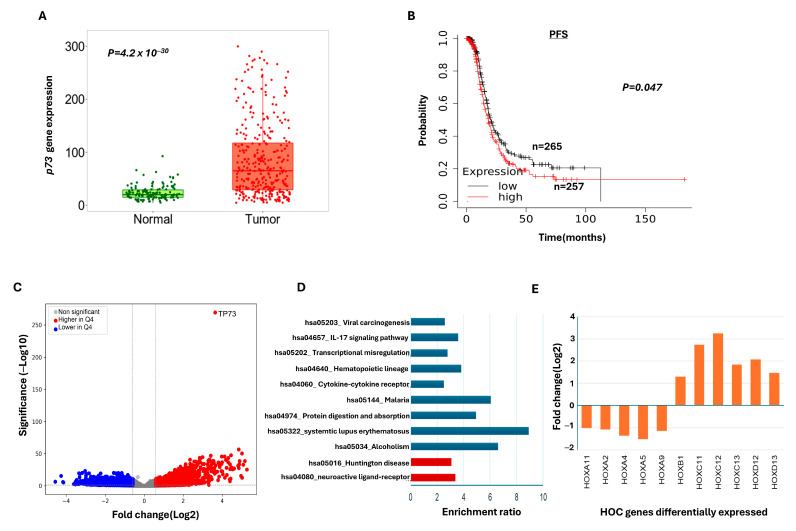
*p73* transcripts in ovarian cancers. (**A**) *TP73* mRNA levels in normal versus ovarian cancer tissue. (**B**) Kaplan–Meier curve for *TP73* mRNA levels and progression-free survival. (**C**) Volcano plot showing the genes that are expressed at significantly higher (red spots) or lower (blue spots) levels in high-*TP73* tumours (Q4) compared to low-*TP73* tumours (Q1). Significance stated as FDR-corrected *p*-value < 0.05, log2 fold change ≥ 1. (**D**) KEGG pathway enrichment plot showing significant pathways (FDR *p* < 0.05) for genes expressed at higher (red bars) and lower levels (blue bars) in high-*TP73* tumours. (**E**) The significantly differentially expressed HOX genes shown to be expressed at lower or higher levels in relation to high-*TP73* tumours. Differential expression of *TP73* mRNA in normal versus serous cystadenocarcinoma was evaluated using a publicly available database (TNMplot.com). For detailed TCGA bioinformatics analysis, please see Section 4.15.

We evaluated the differential expression of *p73* mRNA in normal versus serous cystadenocarcinoma. As shown in Figure 2A, *p73* transcripts were significantly higher in tumour tissue compared to normal tissue. We then proceeded to conduct a survival analysis on the ovarian cancer genome atlas data (TCGA) using the publicly available dataset at kmplot.com [15]. A higher expression of *p73* was associated with poor PFS compared to tumours with low *TP73* (Figure 2B). *p73* expression did not influence OS in the TCGA cohort (Supplementary Figure S2B).

### 2.4. Bioinformatics

The p73 transcription factor is involved in the regulation of genomic instability, pro-proliferative signalling, the activation of invasion and metastasis, angiogenesis, immune evasion, and altered cellular energetics. To investigate if low or high p73 would alter global gene expression profiles and influence biological behaviour, we proceeded to bioinformatic analysis of the TCGA cohort.

To examine if there were any genetic alterations in *TP73*, we utilised cBioportal to examine the TCGA cohort. When examining genetic alterations and mRNA levels (182 samples), no mutations were identified. We then focused on copy number alterations (CNA; 579 samples), with 5% of samples having CNAs, of which 4% were amplifications and 1% were deletions (Supplementary Figure S3A). There was no significant correlation between CNA and mRNA levels (300 samples), showing that within diploid samples the levels of *TP73* greatly varied (Supplementary Figure S3B).

Next, we investigated the differential gene expression between tumours with low and high *TP73* mRNA expression (TCGA-OV cohort n = 379). The differential analysis between quartile 1 (Q1; low *TP73* expression) and quartile 4 (Q4; high *TP73* mRNA expression) identified 1243 genes expressed at higher levels and 1324 genes expressed at lower levels in high-*TP73* tumours (Q4, log2 fold change ≥ 1 and FDR *p*-value < 0.05; Figure 2C, Supplementary Data S1). *TP73* had log2 fold change of 3.64, showing a significant difference between Q1 and Q4. Pathway analysis identified significant pathways linked with the hsa04080 neuroactive ligand–receptor interaction, and neural genes were expressed at higher levels in Q4 (Figure 2D). The pathways that were identified in genes expressed at lower levels in Q4 were involved in IL7 signalling and transcriptional mis-regulation, including IL6 and histone genes (Figure 2D).

Interestingly, HOX genes were differentially expressed in high-*TP73* tumours, with HOXA genes expressed at lower levels and HOXC and D genes expressed at higher levels (Figure 2E, Supplementary Data S2). HOXC and D genes have previously been shown to predict poor clinical outcomes, and other HOXB genes are linked with chemoresistance [16]. HOTAIR was also expressed at a higher level (log 2-fold change −2.26, FDR *p*-value < 0.05) in high-*TP73* tumours and has been linked to chemoresistance in ovarian cancers [17].

Taken together, the data at the protein and transcriptional level provide evidence that *TP73* is frequently overexpressed in ovarian cancer and is associated with an aggressive platinum-resistant phenotype. To investigate this possibility further, we proceeded to laboratory cell line investigations.

### 2.5. p73 Expression Is Higher in Platinum-Resistant Ovarian Cancer Cells and Is Induced upon Cisplatin Treatment

**Figure 3:**
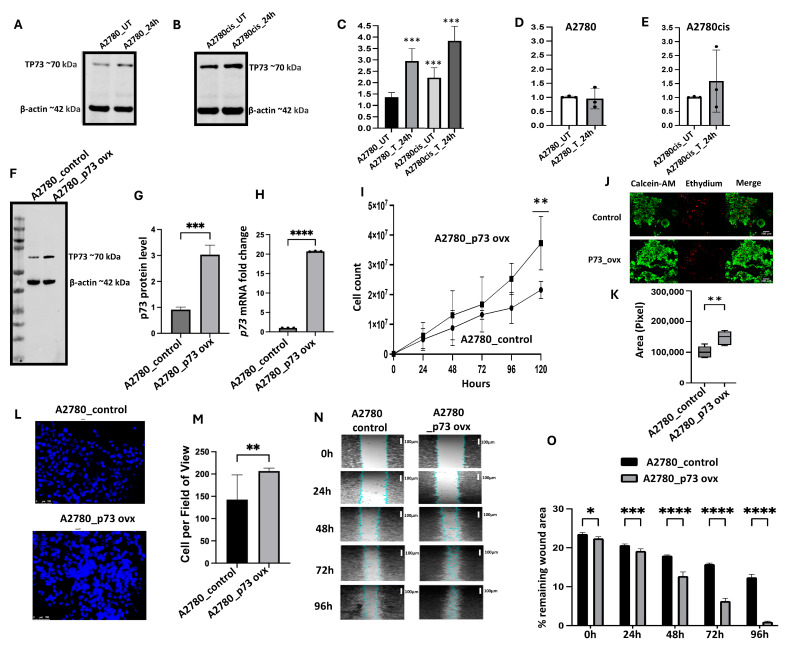
p73 overexpression in ovarian cancer cells. (**A**) Western blot showing p73 expression in A2780 cells at baseline and after 24 h of cisplatin treatment. (**B**) Western blot showing p73 expression in A2780cis cells at baseline and after 24 h of cisplatin treatment. (**C**) p73 protein quantification in A2780 and A2780cis cells. (**D**) *TP73* transcript levels at baseline and after 24 h of cisplatin treatment in A2780 cells. (**E**) *TP73* transcript levels at baseline and after 24 h of cisplatin treatment in A2780cis cells. (**F**) Western blot showing stable overexpression of p73 and (**G**) p73 protein quantification in A2780 control cells compared to A2780_p73 ovx cells. (**H**) *p73* m RNA expression in A2780 control cells compared to A2780_p73 ovx cells. (**I**) Cell doubling time assay results for A2780 control cells compared to A2780_p73 ovx cells. (**J**) Three-dimensional spheroid-forming ability of A2780 control cells and A2780_p73 ovx cells. (**K**) Quantification of spheroid size in A2780 control cells compared to A2780_p73 ovx cells. (**L**) Photomicrograph of invasion assay results for A2780 control cells compared to A2780_p73 ovx cells. (**M**) Quantification of invasion in A2780 control cells compared to A2780_p73 ovx cells. (**N**) Photomicrograph of migration assay in A2780 control cells compared to A2780_p73 ovx cells. (**O**) Quantification of migration in A2780 control cells compared to A2780_p73 ovx cells. ‘*’—*p* ≤ 0.05, ‘**’—*p* ≤ 0.01, ‘***’—*p* ≤ 0.001, ‘****’—*p* ≤ 0.0001. All figures are representative of 3 or more experiments. Error bars represent the standard error of mean between experiments. Statistical analyses were performed using GraphPad Prism, version 9. Student’s *t*-test for Western blot and clonogenic assay. One-way ANOVA for yH2AX, 3D, invasion, and migration analyses. Two-way ANOVA for cell cycle analysis and Annexin V analysis.

The laboratory cell line A2780, derived from a patient with previously untreated ovarian cancer, is platinum-sensitive, whereas A2780cis is a platinum-resistant ovarian cancer cell line developed by chronic exposure of the parental cisplatin-sensitive A2780 cell line to increasing concentrations of cisplatin [18]. As shown in Figure 3A,B, basal levels of p73 were high in A2780cis cells compared to A2780 cells. After 24 h of cisplatin treatment, although we observed an increase in p73 level in A2780 cells, p73 expression was significantly higher in A2780cis cells (Figure 3C). Similarly, after 24 h of cisplatin treatment, we observed a significant induction of *TP73* transcripts in A2780cis compared A2780 cells (Figure 3D,E). Taken together, these data suggest that p73 may be overexpressed in platinum-resistant cells compared to platinum-sensitive cells, and this observation concurs with the clinical data presented above.

### 2.6. p73 Overexpression Promotes Aggressive Phenotype in Ovarian Cancer Cells

We then proceeded to induce p73 overexpression via the stable transfection of HA-p73α-pcDNA3 into A2780 cells (Figure 3F,G). We also confirmed *TP73* transcripts to be significantly higher in A2780_p73ovx compared to A2780 control cells (Figure 3H). The cell doubling time assay revealed that A2780_p73ovx cells were significantly enhanced for proliferation compared to the A2780 control cells (Figure 3I). In addition, the spheroid-forming ability of A2780_p73ovx cells was significantly enhanced compared to the A2780 control cells (Figure 3J,K). A2780_p73ovx cells were also more invasive (Figure 3L,M) and migratory (Figure 3N,O) than A2780 control cells.

### 2.7. p73 Overexpression Leads to Platinum Resistance in Ovarian Cancer Cells

**Figure 4:**
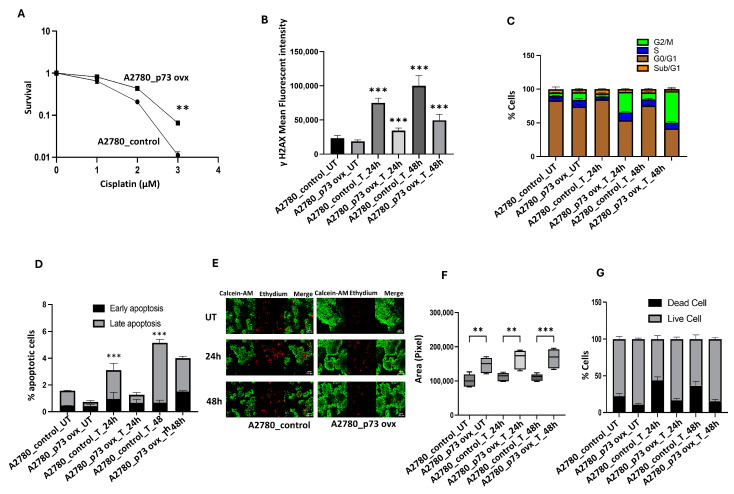
p73 overexpression and cisplatin sensitivity. (**A**) Cisplatin sensitivity assessed by clonogenic survival assay in A2780 control cells compared to A2780_p73 ovx cells. (**B**) Quantification of γH2AX nuclear fluorescence by ImageJ software [UT = untreated, T = cisplatin-treated]. (**C**) Cell cycle analysis (via flow cytometry) of A2780 control cells and A2780_p73 ovx cells treated with cisplatin for 24 h or 48 h [UT = untreated, T = cisplatin-treated]. (**D**) Annexin V analysis for apoptotic cells in A2780 control cells and A2780_p73 ovx cells treated with cisplatin for 24 h or 48 h [UT = untreated, T = cisplatin-treated]. (**E**) Representative photomicrographic images of A2780 control cells and A2780_p73 ovx 3D spheroids treated with cisplatin for 24 h or 48 h [UT = untreated, T = cisplatin-treated]. (**F**) Quantification of spheroid size (via ImageJ software) of A2780 control cells and A2780_p73 ovx 3D spheroids treated with cisplatin for 24 h or 48 h [UT = untreated, T = cisplatin-treated]. (**G**) Quantification of spheroid cell viability (via flow cytometry) of A2780 control cells and A2780_p73 ovx 3D spheroids treated with cisplatin for 24 h or 48 h [UT = untreated, T = cisplatin-treated]. ‘**’—*p* ≤ 0.01, ‘***’—*p* ≤ 0.001. All figures are representative of 3 or more experiments. Error bars represent standard error of the mean between experiments. Statistical analyses were performed using GraphPad Prism, version 9. Student’s t-test for clonogenic assay. One-way ANOVA for yH2AX analysis. Two-way ANOVA for cell cycle analysis and Annexin V analysis.

As shown in Figure 4A, the clonogenic assay showed that A2780_p73ovx cells are platinum-resistant compared to A2780 cells. As γH2AX accumulation is a marker of DNA double-strand break accumulation, we treated A2780_p73ovx and A2780 control cells with 1 µM of cisplatin and monitored γH2AX accumulation at baseline and after 24 h and 48 h of cisplatin treatment. The A2780 control cells showed a significant accumulation of γH2AX compared to A2780_p73ovx cells (Figure 4B). These data suggest increased DNA repair capacity in A2780_p73ovx cells compared to the platinum-sensitive A2780 control cells. p73 is known to have cell cycle regulatory roles at the G1, G2-M, and M checkpoints. We therefore monitored cell cycle progression upon 1 µM of cisplatin treatment. Compared to untreated A2780 cells, the fraction of cells in the G2-M phase increased in untreated A2780_p73ovx cells (Figure 4C). Following 1 µM of cisplatin treatment, we observed a significant accumulation of A2780_p73ovx cells in G2-M compared to A2780 control cells at 24 h and 48 h (Figure 4C). There were increased apoptotic A2780 cells following 1 µM of cisplatin treatment at 24 h and 48 h (Figure 4D). However, the accumulation of apoptotic cells was significantly less in A2780_p73ovx cells (Figure 4D). Moreover, A2780 spheroids were sensitive to 1µM of cisplatin treatment, with a progressive accumulation of dead cells at 24 h and 48 h (Figure 4E–G). In contrast, A2780_p73ovx spheroids remain resistant to 1 µM of cisplatin treatment (Figure 4E–G). Taken together, these data provide pre-clinical evidence that p73 overexpression is associated with platinum resistance.

### 2.8. p73 Overexpression Is Associated with DNA Repair Gene Upregulation

**Figure 5:**
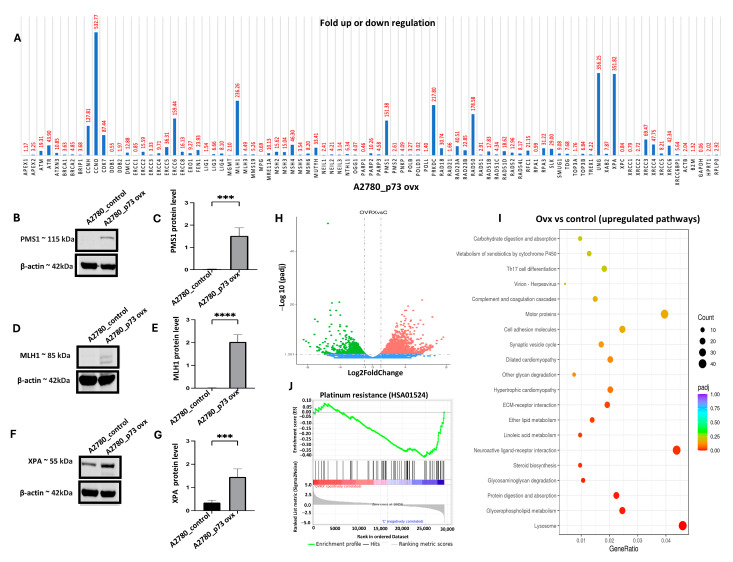
p73 overexpression, DNA repair, and transcriptomic expression profiling. (**A**) Real-time PCR analysis using RT^2^ Profiler PCR Array to evaluate expression of 84 genes involved in DNA damage signalling and repair in A2780 and A2780_p73ovx cells. (**B**) Western blot showing expression of PMS1 protein expression in A2780 control cells and A2780_p73 ovx cells. (**C**) Quantification of PMS1 protein expression in A2780 control cells and A2780_p73 ovx cells. (**D**) Western blot showing expression of MLH1 protein expression in A2780 control cells and A2780_p73 ovx cells. (**E**) Quantification of MLH1 protein expression in A2780 control cells and A2780_p73 ovx cells. (**F**) Western blot showing expression of XPA protein expression in A2780 control cells and A2780_p73 ovx cells. (**G**) Quantification of XPA protein expression in A2780 control cells and A2780_p73 ovx cells. (**H**) Volcano plot showing the genes that are expressed at significantly higher (red spots) or lower (blue spots) levels in A2780_p73 ovx cells compared to A2780 control cells. (**I**) KEGG pathway enrichment plot showing significantly upregulated pathways (FDR *p* < 0.05) in A2780_p73 ovx cells compared to A2780 control cells. (**J**) Gene set enrichment analysis (GSEA) of markers of platinum resistance in A2780_p73 ovx cells compared to A2780 control cells. ‘***’—*p* ≤ 0.001, ‘****’—*p* ≤ 0.0001. All figures are representative of 3 or more experiments. Statistical analyses were performed using GraphPad Prism, version 9. Student’s *t*-test for Western blots. RNA-seq analysis was performed on A2780_control and A2780_p73-overexpressed cells using Novogene. Please see Section 4.16 for full details.

p73 belongs to the p53 family of transcription factors. We hypothesised that p73 overexpression may result in the transcriptional upregulation of DNA repair genes involved in the repair of platinum-induced DNA damage. To address this possibility, real-time PCR was performed using an RT^2^ Profiler PCR Array to evaluate the expression of 84 genes involved in DNA damage signalling and repair in A2780 and A2780_p73ovx cells. As shown in Figure 5A, we observed the upregulation of several genes involved in DNA damage signalling and response (*ATM*, *ATR*, *MRE11*, *PRKDC*, and *RAD50*), mismatch repair (*MLH1*, *MSH2*, *MSH3*, *MSH4*, and *PMS1*), nucleotide excision repair (XPA, ERCC1, ERCC4, ERCC5, ERCC6, *RAD23A,* and *RAD18*), homologous recombination (XRCC3, *RAD51B*, and *RAD51D*) non-homologous end joining (XRCC4 and XRCC6), and base excision repair (UNG) in A2780_p73ovx cells compared to A2780 control cells. Using a panel of antibodies, we also confirmed the overexpression of PMS1 (Figure 5B,C), MLH1 (Figure 5D,E), and XPA (Figure 5F,G) in A2780_p73ovx cells compared to A2780 control cells. Together, these data suggest that the upregulation of multiple DNA repair pathway genes in p73-overexpressed cells may contribute to platinum resistance.

### 2.9. RNA-Seq Analysis

p73 is not only involved in genomic instability, but also in pro-proliferative signalling, the evasion of growth suppression, the activation of invasion, metastasis, angiogenesis, immune evasion, altered cellular energetics, neo-neurogenesis, and response to cytotoxic therapy. We therefore conducted global gene expression analysis on A2780_p73ovx cells and A2780 control cells. Differential genes were identified between A2780_p73ovx cells and A2780 control cells. There were 1827 genes downregulated and 2693 genes upregulated in A2780_p73ovx cells (Figure 5H, Supplementary Data S3). Pathway analysis using KEGG pathways identified several significantly upregulated genes; the pathways included metabolism pathways and ECM–receptor interactions (Figure 5I). Downregulated pathways included the PI3K-AKT pathway, cellular senescence, and cell cycle (Supplementary Figure S4A). Gene set enrichment analysis (GSEA) revealed significantly enriched genes involved in platinum resistance in A2780_p73ovx cells compared to A2780 cells (Figure 5J, Supplementary Figure S4B, Supplementary Data S4).

Taken together, these clinical and pre-clinical data provide evidence that p73 overexpression influences platinum resistance. To validate this observation further, we proceeded to deplete p73 in platinum-resistant A2780cis cells and investigate platinum sensitivity.

### 2.10. p73 Depletion in A2780cis Cells Promotes Cisplatin Sensitivity

**Figure 6:**
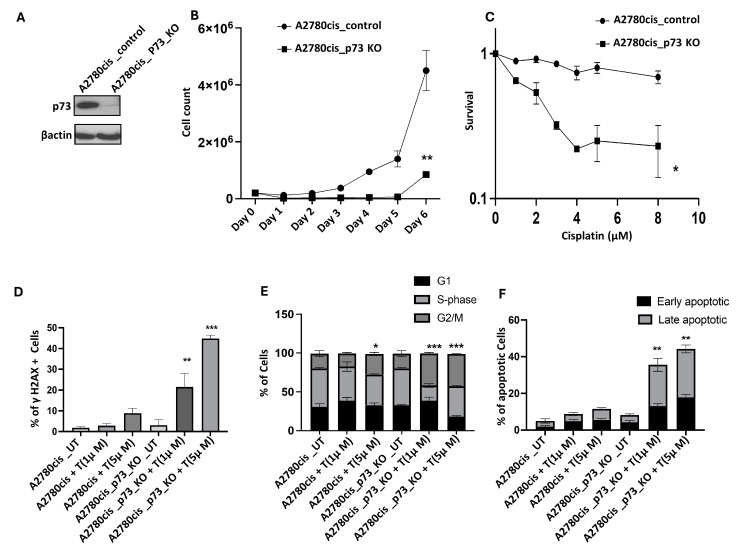
p73 depletion and cisplatin sensitivity. (**A**) Western blot showing p73 depletion in A2780cis_KO cells compared to A2780cis_control cells. (**B**). Cell doubling time assay results for A2780cis_KO cells compared to A2780cis _control cells. (**C**) Cisplatin sensitivity (assessed by clonogenic survival assay) in A2780cis_KO cells compared to A2780cis _control cells. (**D**) Quantification of γH2AX nuclear fluorescence (via ImageJ software) in A2780cis_KO cells and A2780cis _control cells treated with cisplatin [UT = untreated, T = cisplatin-treated]. (**E**) Cell cycle analysis (via flow cytometry) of A2780cis_KO cells and A2780cis _control cells treated with cisplatin [UT = untreated, T = cisplatin-treated]. (**F**) Annexin V analysis of apoptotic cells in A2780cis_KO cells and A2780cis _control cells treated with cisplatin [UT = untreated, T = cisplatin-treated]. ‘*’—*p* ≤ 0.05, ‘**’—*p* ≤ 0.01, ‘***’—*p* ≤ 0.001. All figures are representative of 3 or more experiments. Statistical analyses were performed using GraphPad Prism, version 9. Student’s *t*-test for Western blots. One-way ANOVA for yH2AX analysis. Two-way ANOVA for cell cycle analysis and Annexin V analysis.

We generated p73 knockout (KO) from platinum-resistant A2780cis cells using the CRISPR/Cas-9 system (Figure 6A). A2780cis_p73 KO cells proliferate slowly compared to A2780cis control cells (Figure 6B) and are cisplatin-sensitive (Figure 6C). Following 1µM or 5µM of cisplatin treatment, we observed increased γH2AX accumulation (Figure 6D), G2M or S and G2-M accumulation (Figure 6E), and increased apoptosis (Figure 6F) in A2780cis_p73 KO cells compared to A2780cis control cells. To validate this further, we generated HEK293_p73_KO cells (Supplementary Figure S5A) and observed reduced proliferation (Supplementary Figure S5B) and increased sensitivity to cisplatin (Supplementary Figure S5C) compared to the control cells. Taken together, these data provide evidence that p73 depletion leads to cisplatin sensitivity.

## 3. Discussion

p73 is a member of the p53 family of transcription factors, and has pleiotropic functions during neurodevelopment, tissue homeostasis, and cancer pathogenesis [1,2,3,4,5,6,7,8,9]. The clinicopathological and functional significance of p73 is largely unknown in ovarian cancers. Here, we show that p73 overexpression is associated with aggressive phenotypes, including high grade, advanced-stage disease, and shorter PFS. Significantly higher levels of *TP73* transcripts were also observed in tumour compared to normal tissue and linked with shorter PFS. Preclinically, p73 overexpression in platinum-sensitive A2780 cells increased proliferation, invasion, spheroid-forming ability, DNA repair capacity, and the upregulation of multiple genes involved in DNA repair and platinum resistance. In contrast, p73 deletion in platinum-resistant A2780cis leads to reduced proliferation and enhanced sensitivity to cisplatin, along with DNA double-strand break accumulation, G2/M cell cycle arrest, and increased apoptosis.

Previous studies have indicated a role for p73 in ovarian cancer pathogenesis. Ng et al., showed the increased expression of p73 in a panel of ovarian cancer cell lines and human tumours [19]. In another study, elevated levels of *TP73* mRNA splice variants and p73 protein were observed in invasive cancers compared to ovarian adenomas [20]. However, clinicopathological associations were not described in the above studies. In another study of 100 ovarian tumours, trans-dominant DeltaTAp73 isoforms, which can epigenetically inhibit p53, were frequently upregulated and associated with aggressive ovarian cancers [21]. In a subsequent study, the investigators showed that trans-dominant DeltaTAp73 isoforms contribute to cisplatin resistance, particularly in p53-mutant cancers [13]. In the current study, we show that tumours with wild-type p53 and a low level of p73 expression have good PFS, indicating platinum sensitivity. p73 overexpression has also been described in other solid tumours, including liver, bladder, prostate, and colorectal cancers [1,5,9]. In contrast, a loss of p73 has been shown in pancreatic cancers [22]. Several isoforms can be transcribed from the p73 locus. It has previously been shown that the ratio between TA and δN splice variant expression could influence biology and prognosis. Accordingly, δNp73 overexpression is correlated with aggressive features and poor prognosis in neuroblastoma, prostate, head and neck, and cervical cancers [1,5,9]. A limitation of our study is that we did not investigate individual TP73 splice variants due to the non-availability of antibody clones that recognised specific TP73 splice variants. However, we used a rabbit monoclonal anti-TP73 antibody (Abcam clone-ab189896) for the IHC studies. The antibody has been shown by the manufacturer (Product datasheet: Anti-p73 antibody [EPR18409(T)(MIX)] ab189896) to recognise C-terminal fragments of p73 containing amino acids 380–636. These data imply that the antibody recognizes all splice variants, and the levels indicate the total p73 expression in cells. The protein levels of p73 and its mRNA levels could have a high disagreement due to several known mechanisms of post-translational regulation. A limitation of our study is that we were unable to investigate this possibility due to the unavailability of mRNA expression data in the cohort upon which immunohistochemical analysis was performed in the current study.

p53 mutation can lead to p53 stability and accumulation in cells. Accordingly, p53 overexpression is a surrogate marker of p53 mutation status in tumours [23]. Our observation that p53 overexpression is associated with aggressive phenotypes and poor PFS concurs with previous clinical data [24]. We also show that p53-overexpressing tumours with high levels of p73 have worse PFS compared to wild-type p53 with low p73 expression. Moreover, in p53 wild-type tumours, the presence of p73 overexpression adversely influences clinical outcome. These data suggest that the crosstalk between p53 and p73 could considerably influence ovarian cancer pathogenesis. However, detailed mechanistic studies will be required to confirm our hypothesis. The bioinformatics data presented here also suggest that p73 overexpression could influence whole-genome expression and promote an aggressive phenotype.

Preclinically, p73 overexpression in A2780 cells increased proliferation, invasion, spheroid-forming ability, and DNA repair capacity, associated with the upregulation of multiple DNA repair gene expression and platinum resistance. Increased protein levels of PMS1, MLH1, and XPA may contribute to platinum resistance, but detailed functional studies will be required to confirm this hypothesis. In contrast, p73 deletion in A2780cis leads to reduced proliferation and enhanced sensitivity to cisplatin, along with DNA double-strand break accumulation, G2/M cell cycle arrest, and increased apoptosis. A limitation of this study is that we were unable to evaluate DNA repair gene expression and conduct DNA repair protein expression studies in p73-depleted cells compared to the control. Future detailed functional studies will be required to clarify the role of p73 in the transcriptional regulation of DNA repair genes.

## 4. Materials and Methods

### 4.1. Patients

The expression of p73 was evaluated on tissue microarrays of 331 consecutive epithelial ovarian cancers treated at Nottingham University Hospitals (NUH), Nottingham, UK, between 1997 and 2010. Patients received primary debulking surgery. Patients were comprehensively staged as per the International Federation of Obstetricians and Gynaecologists (FIGO) Staging System for Ovarian Cancer. Overall survival was calculated from the operation date until the time of death or the last date of follow-up, when any remaining survivors were censored. All patients received platinum-based chemotherapy. Platinum resistance was defined as patients who had progression during first-line platinum chemotherapy or relapse within 6 months after the completion of chemotherapy. Progression-free survival was calculated from the date of the initial surgery to disease progression or from the date of the initial surgery to the last date known to be progression-free for those censored. Patient demographics are summarised in Supplementary Table S1.

Tumour Marker Prognostic Studies (REMARK) criteria, recommended by McShane et al. [25], were followed throughout this study. This study was carried out in accordance with the declaration of Helsinki, and ethical approval was obtained from the Nottingham Research Ethics Committee (REC Approval Number 06/Q240/153).

### 4.2. Tissue Microarray (TMA) and Immunohistochemistry (IHC)

Tumour samples were arrayed in tissue microarrays (TMAs) constructed with 2 replicate 0.6 mm cores from the tumours. Immunohistochemical staining was performed using the Thermo Fisher Scientific (Biohub, Cheshire, UK) Shandon Sequenza chamber system (REF: 72110017, Biohub, Cheshire, UK) in combination with the Novolink Max Polymer Detection System (RE7280-K: 1250 tests, Buffalo Grove, IL, USA) and Leica Bond Primary Antibody Diluent (AR9352, Buffalo Grove, IL, USA), each used according to the manufacturer’s instructions (Leica Microsystems, Buffalo Grove, IL, USA). The TMA slides were deparaffinized with xylene and then rehydrated through five decreasing concentrations of alcohol (100%, 90%, 70%, 50%, and 30%) for two minutes each. Pre-treatment antigen retrieval was carried out on the TMA sections using sodium citrate buffer (pH 6.0) and heated at 95 C in a microwave (Whirlpool JT359 Jet Chef 1000W, Peterborough, UK) for 20 min. A set of slides were incubated for 1 h at room temperature with the primary rabbit monoclonal anti-p73 antibody (AB189896, Abcam, Cambridge, Cambridgeshire, UK) at a dilution of 1:500. Sections were counterstained with haematoxylin. Monoclonal mouse anti-human p53 [clone DO7, cell signalling] was used and was diluted at 1:100 in Leica antibody diluent (RE AR9352, Leica, Biosystems, Newcastle Upon Tyne, Tyne and Wear, UK) and incubated for 30 min at room temperature. Immunostaining for p53 showed only nuclear expression. p53 was evaluated using a semi-quantitative system. H-score from 0 to 300 was calculated for each case.

Cases with multiple cores were scored and the average was used as the final score. Negative (by omission of the primary antibody and IgG-matched serum) and positive controls (lymph node/spleen) were included in each run. Not all cores within the TMA were included for IHC analysis due to missing cores or the absence of tumour cells.

### 4.3. Evaluation of Immune Staining

The cores of TMA were assessed for suitability of scoring. For example, cores with less than 20% tumour were excluded from the study. For each sample, a visual assessment of the staining was performed, and the subcellular localization of each marker was identified (nuclear, cytoplasm, cell membrane, or mixed). Intensities of subcellular localisation were evaluated for each marker as follows: 0 = no staining, 1 = weak staining, 2 = moderate staining, 3 = strong staining. The percentage of protein expression was evaluated (0–100%). In addition, the histochemical score (H-score) (range 0–300) was calculated by multiplying the intensity of staining and the percentage of staining. Not all cores within the TMA were included for IHC analysis due to missing cores or the absence of tumour cells. X-tile bioinformatics software version 3.6.1 (School of Medicine, Yale University, New Haven, CT, USA) was used to generate the best cut-offs for both the nuclear and cytoplasmic expression of each marker based on patient outcomes. Low nuclear p73 was defined as an H-score below 70 and low cytoplasmic p73 was defined as an H-score below 40. H-scores ≥ 4 were considered p53-positive (mutant) tumours.

### 4.4. Statistical Analysis

Statistical Package for the Social Sciences software v.27.0 (SPSS, Chicago, IL, USA) was used for statistical analysis. The correlation between clinical and pathological characteristics using categorized data was calculated using Chi-square tests. All tests were 2-tailed. Survival rates were determined using the Kaplan–Meier method and compared with the log-rank test. A *p*-value < 0.05 was identified as statistically significant.

### 4.5. TP73 mRNA Expression in Ovarian Cancers

The differential expression of *TP73* mRNA in normal versus serous cystadenocarcinoma was evaluated using a publicly available database (TNMplot.com) [26]. The prognostic or predictive significance of *TP73* mRNA was investigated in the ovarian cancer genome atlas data (TCGA) cohort using the publicly available dataset at kmplot.com [15].

### 4.6. TCGA Bioinformatics Analysis

Analysis of the *TP73* genetic alterations and mRNA levels of the TCGA-OV specimens (TCGA Firehose Legacy, 182 samples) was performed using cBioportal [27]. As no mutations were seen, removing the mutation data increased the tumour sample size to 579 samples for copy number alterations, of which 300 samples had matching mRNA-level data. For the differential analysis of tumours with high versus low *TP73*, TCGA ovarian cancer (TCGA, [28]) RNAseq expression data for 379 samples was obtained from GDC (https://portal.gdc.cancer.gov/). The TCGA-OV specimens were ranked from the lowest to highest expression of *TP73* and quartiles (Q1–4) were calculated. Using DESeq2, a comparison between Q1 and Q4 was performed to obtain the differentially expressed genes between the low and high levels of *TP73* [29]. WebGestalt v2019 was used to identify significant KEGG pathways (FDR-*p* value < 0.05) using differential genes with a log2 fold of 1 and above (FDR-*p* value < 0.05) [30].

### 4.7. Cell Lines and Tissue Culture

Laboratory cell lines A2780 (platinum-sensitive) and A2780cis (platinum-resistant) were purchased from Sigma Aldrich (Gillingham, UK). Laboratory cell lines PE01 (BRCA2-deficient, platinum-sensitive) and PE04 (BRCA2-proficient, platinum-resistant), and HEK293T kidney embryonic cells were purchased from the American Type Culture Collection (ATCC, Manassas, VA, USA). Cells were cultured in RPMI (R8758, Merck, Gillingham, Dorset, UK) supplemented with 10% FBS (F4135, Merck, UK) and 1% penicillin–streptomycin (P4333, Merck, UK). HEK293T cells were cultured in Dulbecco’s Modified Eagle Medium supplemented with 10% FBS and 1% penicillin–streptomycin (Thermofisher Scientific, Biohub, Cheshire, UK).

### 4.8. Western Blot Analysis

Cells were harvested and lysed in RIPA buffer (R0278, Sigma, Welwyn Garden City Hertfordshire, UK) with the addition of protease cocktail inhibitor (P8348, Sigma, UK), phosphatase inhibitor cocktail 2 (P5726, Sigma, UK), and phosphatase inhibitor cocktail 3 (P0044, Sigma, UK), and stored at −20 °C. Proteins were quantified using the BCA Protein Assay kit (23225, Thermofisher, UK). Samples were run on SDS-bolt gel (4–12%) bis-tris. Membranes were incubated with primary antibodies as follows: rabbit monoclonal anti-p73 (1:5000) (AB189896, Abcam, UK) at 4 °C overnight, mouse monoclonal anti-p73 [1:5000, Abcam (Ab189896), 4 °C overnight incubation], rabbit monoclonal anti-GAPDH [1:3000, Abcam Ab9485, 1h room temperature], rabbit monoclonal anti-YY1 [1:2000, Abcam Ab109228, 1h room temperature], rabbit monoclonal anti-ERCC6 [1:500, Thermo fischer (PA5-120625), 4 °C overnight incubation], anti-MLH1 [1:500, Thermo fischer (MA5-15431), 4 °C overnight incubation], mouse monoclonal anti-PMS1 [1:1000, Thermo fischer (PA5-86724), 4 °C overnight incubation], rabbit monoclonal anti-XPA [1:2000, Abcam (ab85914), 4 °C overnight incubation]. Membranes then were washed and incubated with rabbit monoclonal anti-β-actin [1:5000, Abcam Ab8226, 1h room temperature]. Membranes were then washed and incubated with infrared dye-labelled secondary antibodies (LiCor, Cambridge, Cambridgeshire, UK) [IRDye 800CW Donkey Anti-Rabbit IgG (926-32213) and IRDye 680CW Donkey Anti-Mouse IgG (926-68072)] at a dilution of 1:10,000 for 1 h. Membranes were scanned with a LiCor Odyssey machine (700 and 800 nm) to determine protein levels.

### 4.9. Stable Transfection of p73 pcDNA (Knock-In) Plasmid

Plasmid HA-p73α-pcDNA3 from Addgene (Cat. 22102, Watertown, MA 02472, USA) containing *TP73* cDNA was used. The transfection cells were seeded in 6-well plates overnight at 60–70% confluency. In the following day’s experiment, 7.5 µL of lipofectamine 3000 was prepared in 500 µL of Opti-MEM medium, along with 2 ug of plasmid dissolved in 500 µL of Opti-MEM medium and P3000 reagent. A diluted lipofectamine solution was added to a diluted DNA tube and incubated at room temperature for 15 min. The cells were then washed with Opti-MEM medium, and the transfection mixture was added to the plates and incubated overnight at 37 °C in 5% CO_2_/95% air. The transfection medium was changed to a complete culture medium the next day. Following 48 h, Neomycin (selected with G418) was used to isolate the desired clones. A2780_p73_Knock-in cells were selected at 400 µg/mL of G418. Selection doses were determined pre-transfection using the G418 kill curve experiment in ovarian cells. At 10–14 days, A2780_p73_Knock-in cells were maintained at 200 μg/mL of G418. Stable transfected colonies were amplified, and transfection efficiency was determined by Western blotting.

### 4.10. Clonogenic Assays

In the clonogenic assay, 350 cells/well of control and p73-overexpressing cells were seeded in 6-well plates and left at 37 °C in a 5% CO_2_ atmosphere. Cisplatin (kindly provided by Nottingham University Hospital) or mirin (M9948, Sigma, UK) was added at the indicated concentrations and the plates were left at 37 °C in a 5% CO_2_ atmosphere for 14 days. Later, the plates were washed with PBS and stained with methanol, crystal violet, and acetic acid mixture, and the colonies were counted. 

### 4.11. Cell Doubling Time Assay

Next, 1 × 10^5^ cells/well of control and p73-overexpressing cells were seeded in 6-well plates and allowed to grow over five days. On days 1, 2, 3, 4, and 5, cell numbers were counted. Cell doubling was calculated as follows: doubling time (hours) = T multiplied by ln(2) divided by ln(Nt/N0). In this formula, T refers to the incubation duration in hours, Nt represents the final cell count, and N0 is the initial cell count.

### 4.12. Invasion and Migration Assays

For the invasion assay, cells were seeded in the upper chamber of polycarbonate membrane inserts (8 µM pore size) (Cell Biolabs, Exeter, Devon, UK) in serum-free medium and left to invade toward 10% serum-containing medium for 24 h. Then, the medium containing non-invasive cells was aspirated from the inserts and the inner side was washed with distilled water and stained with crystal violet for 10 min. Cells were extracted, and 100 µL from each sample was transferred to a 96-well microtiter plate to measure OD at 560 nm. For the migration assays (Cat#: IB-81176; Thistle Scientific Ltd., Glasgow, UK), cells were seeded in a 96-well plate containing a hydrogel spot non-migratory area and left to adhere overnight. Then, the hydrogel area was digested and cells were left to migrate for 24 h. Then, the wells were washed three times, fixed, and stained as per the manufacturer’s protocol. Cell migration images were analysed with ImageJ software (https://imagej.net/ij/, accessed on 3 June 2023).

### 4.13. Generation of 3D Spheroids

Next, 4 × 10^4^ cells per well were plated in ultra-low-attachment 6-well plates in Promocell serum-free tumour sphere medium (C-28070). Cells were then topped off with fresh medium every three days until spheroid structures were formed. Spheroids were treated with cisplatin for 48 h. To quantify cell viability, the LIVE/DEAD Viability/Cytotoxicity Kit (L3224, Thermo Fisher Scientific) was used. Briefly, the spheroids were collected by trypsinization, washed with PBS, and centrifuged at 1000′g for 5 min. The light-protected cellular pellet in PBS was loaded with 0.1 mM of Calcein-AM and 1 mM of Ethidium homodimer-1 for 20 min at room temperature. The samples were then analysed on a Beckman Coulter FC500 flow cytometer (High Wycombe Buckinghamshire, UK) using a 495 nm laser for excitation and a 515 nm laser for emission data for Calcein-AM (Thermofisher Scientific, Biohub, Cheshire, UK), and a 495 nm laser for excitation and emission at 635 nm for Ethidium Homodimer-1. In addition, Image J software was used to calculate spheroid diameter. The mean of three diagonal diameters was taken as the diameter for each spheroid. At least 10 spheroids were measured.

### 4.14. Functional Studies

Next, 1 × 10^5^ cells per well were seeded in 6-well plates and left overnight at 37 °C in a 5% CO_2_ atmosphere. After 24 h, 1 or 5 µM of cisplatin was added to cells and incubated for 24 h and 48 h. Cells then were collected by trypsinization, washed with ice-cold PBS, and fixed in 70% ethanol for 1 h at −20 °C. After removal of the fixative solution by centrifugation, for DNA double-strand break analysis, cells were stained with 2 mg/mL of phospho-Histone (γH2AX) Ser139 (16202A, Millipore, Livingston, West Lothian, UK). For cell cycle analysis, cells were treated with 20 mg/mL RNase A (12091021, Invitrogen, Paisley, Renfrewshire, UK), and then 10 mg/mL Propidium Iodide (P4170, Sigma Aldrich) was added to determine the cell cycle distribution. The samples were analysed on a Beckman Coulter FC500 flow cytometer using a 488 nm laser for excitation and emission data for PI, collected using a 620 nm bandpass filter (FL3) and a 525 nm bandpass filter (FL1) for FITC-anti-phospho-Histone H2A.X. For the apoptosis assay, cells were analysed using the Annexin V detection kit (556547, BD Biosciences, Wokingham, Berkshire, UK). Briefly, cells were trypsinized and washed with PBS, and the cellular pellet was re-suspended in Annexin Binding Buffer (Invitrogen, Paisley, Renfrewshire, UK) (1x). Then, 2.5 mL of FITC Annexin V and 2.5 mL of Propidium Iodide were added to the cells. After incubation, 300 mL of Annexin Binding Buffer (1x) was added to each tube. Samples were analysed on a Beckman Coulter FC500 flow cytometer. Data were analysed using Flowjo software (https://www.flowjo.com/, accessed on 10 June 2023). Graphical representation was established and statistical analysis was performed using GraphPad Prism 7 (GraphPad, La Jolla, CA, USA).

### 4.15. Real-Time PCR

For DNA repair pathway-focused analysis, RT2 PCR array plates were used (PAHS-042ZC, Qiagen, Manchester, Lancashire, UK) (a list of DNA repair genes evaluated in this assay is shown in Supplementary Table S2). RNA was extracted using the RNeasy Mini kit (74104, Qiagen) and cDNA conversion was carried out using the RT2 first-strand kit (330404, Qiagen), as per the manufacturer’s protocol. Samples were run on an ABI-7500 fast block. The data were analysed as per the manufacturer’s recommendations. Real-time PCR was carried out on an Applied Biosystems 75000 FAST cycler (Thermofisher Scientific, Biohub, Cheshire, UK).

### 4.16. RNA-Seq Analysis

RNA-seq was performed on A2780_control and A2780_p73-overexpressed cells. All experiments were performed in triplicate, and sequences were analysed with Novogene (Cambridge, Cambridgeshire, UK). The raw data were transformed to sequenced reads by CASAVA base recognition (Base Calling). The data underwent QC and adaptor removal. Sequences contained N > 10% and a Qscore over 50% for bases below 5. The alignment was performed with HISAT2 as the reference [31]. Feature counts used for quantification [30] and differential gene analysis were performed using DESeq2 [29] (https://bioconductor.org/packages/release/bioc/html/DESeq2.html, accessed on 1 August 2023) and EdgeR [32] (https://bioconductor.org/packages/release/bioc/html/edgeR.html, accessed on 1 August 2023). KEGG pathway analysis was performed to identify significant pathways in the up and downregulated differentially expressed genes [33]. Rmats (https://rnaseq-mats.sourceforge.io/, accessed on 1 August 2023) was utilised for alternative splicing analysis [34].

### 4.17. CRISPR Knock-Out of p73

gRNA oligonucleotides targeting p73 were designed in Snapgene and cloned in the pX330-gRNA vector provided by Feng Zhang (Addgene plasmid # 42230). The sequences of the gRNAs were as follows: “gRNA-1-p73 (agtccaccgccacctcccc) and gRNA-2-p73 (gaggccggcgtggggaaga)”. For the mutation, the gRNA targeted exon1 of the p73 amplicon, resulting in early deletion. Plasmids with positive clones expressing gRNAs were verified by Sanger sequencing. Cells were plated in 6-well plates overnight, then transfected with plasmids expressing gRNA using the Fugene HD transfection reagent. After 48 h, Cas-9 editing efficiency was verified using the T7E1 assay. Cells were seeded at low density for single-cell colony isolation. The isolated colonies were verified by Western blotting and Sanger sequencing.

### 4.18. Statistical Analysis

Data were analysed using GraphPad Prism 9 software. Where appropriate, Student’s *t* tests, ANOVA one-way tests, and ANOVA two-way tests were performed. Using error bars, the standard error of the mean is represented between experiments. Furthermore, *p*-values were indicated as follows: *p*-value < 0.05 = *, *p*-value < 0.01 = **, and *p*-value < 0.001 = ***.

## 5. Conclusions

In conclusion, our clinical and preclinical data provide evidence that p73 is a predictor of platinum resistance in ovarian cancer. p73-directed stratification may have clinical application in ovarian cancer patients.

## Figures and Tables

**Table 1:** p73 cytoplasmic and nuclear co-expression and clinicopathological variables.

Parameters	p73 Cyt/Nuc Co-Expression				*p* Value
	Cyt−/Nuc−	Cyt+/Nuc−	Nuc+/Cyt+	Nuc+/Cyt−	
	*N* (%)	*N* (%)	*N* (%)	*N* (%)	
Pathology Type					
Serous	86 (48.6%)	62 (78.5%)	5 (50.0%)	5 (41.7%)	<0.0001
Mucinous	35 (19.8%)	3 (3.8%)	0 (0.0%)	3 (25%)	
Endometrioid	21 (11.9%)	9 (11.4%)	2 (20%)	0 (0.0%)	
Clear Cell	16 (9.0%)	1 (1.3%)	2 (20%)	1 (8.3%)	
Other	9 (51.1%)	0 (0.0%)	1 (10%)	3 (25.0%)	
Mixed	10 (5.6%)	4 (5.1%)	0 (0.0%)	0 (0.0%)	
Pathology Grade					
Low	30 (20.0%)	6 (8.2%)	0 (0.0%)	3 (27.3%)	0.075
Med	34 (22.7%)	12 (16.4%)	1 (11.1%)	2 (18.2%)	
High	86 (57.3%)	55 (75.3%)	8 (88.9%)	6 (54.5%)	
Pathology Stage					
1	78 (45.6%)	22 (28.6%)	2 (22.2%)	5 (45.5%)	0.135
2	24 (14.0%)	12 (15.6%)	0 (0.0%)	1 (9.1%)	
3	64 (37.4%)	41 (53.2%)	7 (77.8%)	4 (36.4%)	
4	5 (2.9%)	2 (2.6%)	0 (0.0%)	1 (9.1%)	
Residual Tumour After Surgery					
None	122 (74.8%)	43 (62.3%)	4 (50%)	7 (63.6%)	0.003
<1 cm	12 (7.4%)	14 (20.3%)	1 (12.5)	1 (9.1%)	
1–2 cm	4 (2.5%)	7 (10.1%)	2 (25%)	0 (0.0%)	
>2 cm	25 (15.3%)	5 (7.2%)	1 (12.5%)	3 (27.3%)	

## Data Availability

The data supporting the study can be found in the Supplementary Materials file, and the corresponding author can make any materials available upon request. The aggregate data from the referenced datasets are available from the corresponding author on reasonable request.

## Supplementary Materials

The following supporting information can be downloaded at https://www.mdpi.com/article/10.3390/ijms26073239/s1.
