# Supplementary material for: Transcription Factor p73 Is a Predictor of Platinum Resistance and Promotes Aggressive Epithelial Ovarian Cancers
Source: Int J Mol Sci. 2025 Mar 31;26(7):3239. doi: 10.3390/ijms26073239 (PMC11989448; doi:10.3390/ijms26073239)
Supplement: Supplementary file 1 [file ijms-26-03239-s001.zip › Supplementary material.pdf]

Supplementary Table S1: Patient demographics

**Parameters**	**Number (%)**
Surgical Pathology Type	
Serous	158 (57%)
Mucinous	41 (15%)
Endometriod	32 (12%)
Clear Cell	20 (7%)
Other	13 (4%)
Mixed	14 (5%)
Surgical Pathology Grade	
Low	39 (16%)
Med	49 (20%)
High	155 (64%)
**Surgical Pathology Stage**	
1	107 (40%)
2	37 (14%)
3	116 (43%)
4	8 (3%)
Residual Tumour Following Surgery	
None	176 (70%)
<1cm	28 (11%)
1-2cm	13 (5%)
>2cm	34 (14%)
**Platinum Sensitivity**	
Sensitive	215 (92%)

Resistant	20 (8%)

Supplementary Table S2. List of DNA repair genes.

DNA repair gene name
RPA4
PMS2P2
MSH4
GTF2H4
HUS1
RPA1
PARP1
FANCC
PMS2
RAD9B
RPA2
MSH5
CHEK2
FANCE
NBN
BRCA2
ATM
BRCA1
NEIL3
RPA1
RAD51B
ERCC2
RECQL4
RAD51C
RAD1
TOP3A
WRNIP1
PARG
XPA
RRM1
MBD5
RPA3
PMS2P5
MBD3
RAD54B
N4BP2
UNG
MUTYH
RAD51D
PARP3
NEIL2
RAD50

EXO1
FANCA
BRIP1
SMUG1
POLQ
PMS2
PCNA
PNKP
MGMT
POLB
POLE3
RAD23B
RAD52
RAD23A
APEX1
TOP2A
MSH2
ERCC4
XPG
LIG1
FANCG
POLE2
LIG4
XRCC4
BLM
TP53
PMS1
POLD2
ALKBH2
OGG1
PRKDC
ATP23
NTHL1
XRCC5
GTF2H3
ERCC1
POLH
RAD54L
TOP1
LIG3
XRCC1
XRCC6
REV3L
POLD1

FAN1
POLA1
XPC
NEIL1
FEN1
TDP1
DCLRE1A
FANCM
MBD1
RAD21
RAD51AP1
ATR
MRE11
UNG
RFC1
RBBP8
TP53BP1
CDK7
PMS2P3
PARP4
RAD51
XAB2
HUS1
RAD23A
FANCF
CSNK1D
POLD3
POLE4
POLN
ERCC6
RAD9A
MBD2
RECQL5
ENDOV
TREX2
MLH1
CDK12
WRN
FEN1

Supplementary Table S3: Nuclear p73 and clinicopathological variables.

**Parameters**	*TP73 Nuclear expression*		*p value*
	Low	*High*	
	N (%)	*N (%)*	
**Surgical Pathology Type**			
Serous	148 (57.8%)	10 (45.5%)	**0.028**
Mucinous	38 (14.8%)	3 (13.6%)	
Endometriod	30 (11.7%)	2 (9.1%)	
Clear Cell	17 (6.6%)	3 (13.6%)	
Other	9 (3.5%)	4 (18.2%)	
Mixed	14 (5.5%)	0 (0.0%)	
**Surgical Pathology Grade**			
Low	36 (16.1%)	3 (15%)	0.804
Med	46 (20.6%)	3 (15%)	
High	141 (63.2%)	14 (70%)	
**Surgical Pathology Stage**			
1	100 (40.3%)	7 (35%)	0.508
2	36 (14.5%)	1 (5.0%)	
3	105 (42.3%)	11 (55.0%)	
4	7 (2.8%)	1 (5%)	
**Residual Tumour Following Surgery**			
None	165 (71.1%)	11 (57.9%)	0.487
<1cm	26 (11.2%)	2 (10.5%)	
1-2cm	11 (4.7%)	2 (10.5%)	
>2cm	30 (12.9%)	4 (21.1%)	

Supplementary Table S4: Cytoplasmic p73 and clinicopathological variables.

Parameters	*TP73 Cytoplasm expression*		R^2
	Low	*High*	
	N (%)	*N (%)*	*p value*
**Surgical Pathology Type**			
Serous	91 (48%)	67 (75%)	
Mucinous	38 (20%)	3 (3%)	25.679
Endometriod	21 (11%)	11 (13%)	**<0.0001**
Clear Cell	17 (9%)	3 (3%)	
Other	12 (7%)	1 (1%)	
Mixed	10 (5%)	4 (5%)	
**Surgical Pathology Grade**			
Low	33 (21%)	6 (7%)	10.322
Med	36 (22%)	13 (16%)	**0.006**
High	92 (57%)	63 (77%)	
**Surgical Pathology Stage**			
1	83 (46%)	24 (28%)	9.362
2	25 (14%)	12 (14%)	**0.025**
3	68 (37%)	48 (56%)	
4	6 (3%)	2 (2%)	
**Residual Tumour Following Surgery**			
None	129 (74%)	47 (61%)	20.008
<1cm	13 (8%)	15 (19%)	**<0.0001**
1-2cm	4 (2%)	9 (12%)	
>2cm	28 (16%)	6 (8%)	

Supplementary Tables S5: Nuclear p53 and clinicopathological variables

**Parameters**	*TP53 expression*		
	Low	*High*	
	N (%)	*N (%)*	*p value*
**Surgical Pathology Type**			
Serous	47 (42%)	75 (67%)	20.298
Mucinous	21 (19%)	10 (9%)	**0.001**
Endometriod	18 (16%)	12 (11%)	
Clear Cell	14 (12%)	3 (3%)	
Other	8 (7%)	4 (4%)	
Mixed	5 (4%)	7 (6%)	
**Surgical Pathology Grade**			
Low	23 (26%)	8 (8%)	15.022
Med	20 (22%)	16 (15%)	**0.001**
High	47 (52%)	79 (77%)	
**Surgical Pathology Stage**			
1	49 (47%)	38 (35%)	7.598
2	20 (19%)	14 (13%)	0.055
3	32 (31%)	52 (48%)	
4	3 (3%)	5 (4%)	
**Residual Tumour Following Surgery**			
None	81 (78%)	64 (63%)	7.785
<1cm	10 (10%)	12 (12%)	0.051
1-2cm	5 (5%)	6 (6%)	
>2cm	7 (7%)	19 (19%)	

Supplementary Table S6: Nuclear p53 and p73 co-expression and clinicopathological variables

**Parameters**	*TP53/TP73 co-expression*				
	TP53-/ TP73 -	*TP53 + /TP73 -*	*TP53 + / TP73 +*	*TP53 - / TP73 +*	
	N (%)	*N (%)*	*N (%)*	*N (%)*	*p value*
**Surgical Pathology Type**					
Serous	22 (37%)	32 (64%)	27 (79%)	13 (76%)	30.209
Mucinous	14 (23%)	7 (14%)	1 (3%)	1 (6%)	**0.011**
Endometriod	9 (15%)	2 (4%)	5 (15%)	1 (6%)	
Clear Cell	6 (10%)	2 (4%)	0 (0%)	1 (6%)	
Other	6 (10%)	3 (6%)	0 (0%)	0 (0%)	
Mixed	3 (5%)	4 (8%)	1 (3%)	1 (6%)	
**Surgical Pathology Grade**					
Low	14 (30%)	5 (11%)	2 (6%)	1 (7%)	12.795
Med	8 (18%)	10 (21%)	5 (16%)	2 (13%)	**0.046**
High	24 (52%)	32 (68%)	25 (78%)	12 (80%)	
**Surgical Pathology Stage**					
1	25 (45%)	19 (39%)	10 (30%)	3 (19%)	8.235
2	11 (20%)	7 (14%)	5 (15%)	3 (19%)	0.511
3	18 (32%)	20 (41%)	17 (52%)	10 (62%)	
4	2 (3%)	3 (6%)	1 (3%)	0 (0%)	
**Residual Tumour Following Surgery**					
None	44 (82%)	29 (62%)	17 (59%)	10 (67%)	23.375
<1cm	5 (9%)	3 (6%)	5 (17%)	3 (20%)	**0.005**
1-2cm	0 (0%)	1 (2%)	3 (10%)	2 (13%)	

>2cm	5 (9%)	14 (30%)	4 (14%)	0 (0%)

Supplementary Figure legends

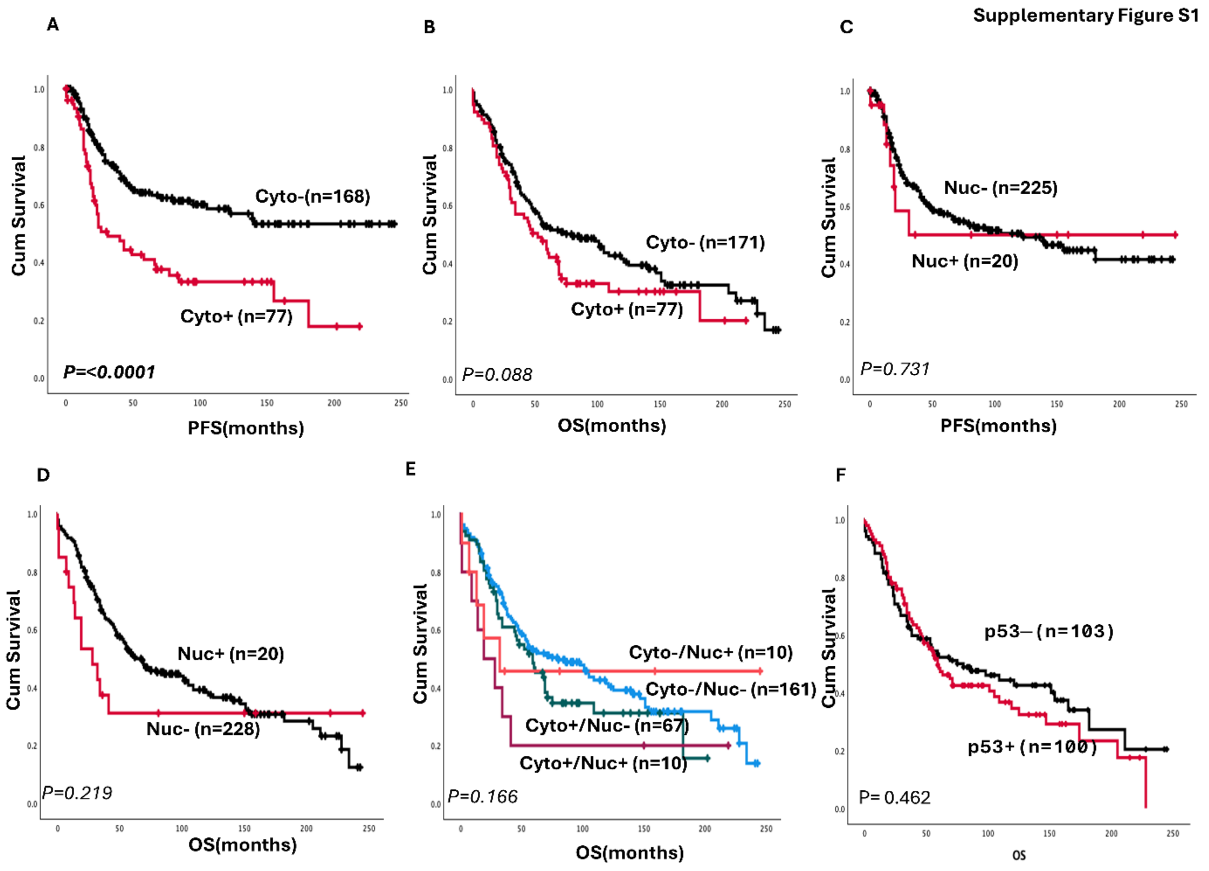

Supplementary Figure S1: (A) Kaplan-Meier curve for p73 cytoplasmic expression and progression free survival (PFS) **(B)** Kaplan-Meier curve for p73 cytoplasmic expression and overall survival (OS) **(C)** Kaplan-Meier curve for p73 nuclear expression and progression free survival (PFS). **(D)** Kaplan–Meier curve for Kaplan-Meier curve for p73 nuclear expression and OS. **(E)** Kaplan-Meier curve for p73 nuclear/cytoplasmic co-expression and OS. **(F)** Kaplan–Meier curve for Kaplan-Meier curve for p53 nuclear expression and OS.

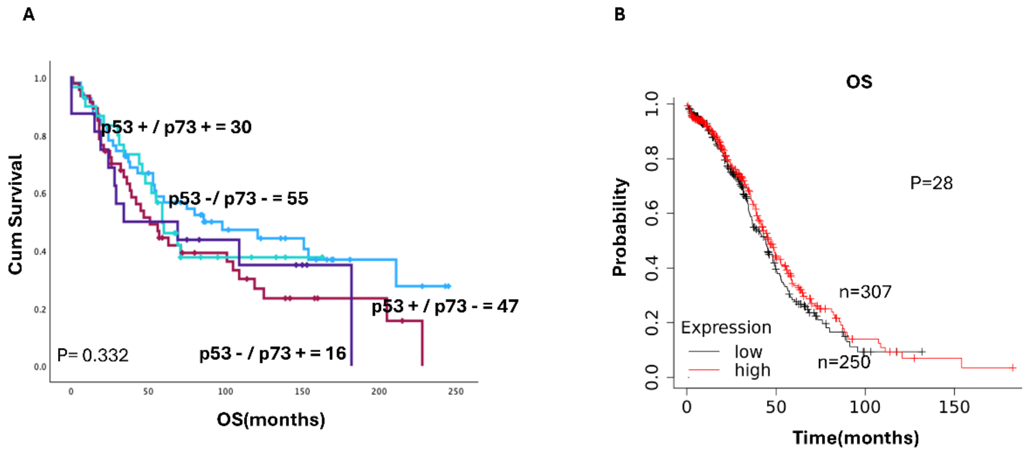

Supplementary Figure S2: (A) Kaplan-Meier curve for p73 nuclear/cytoplasmic co-expression and overall survival (OS). **(b)** Kaplan-Meier curve for *p73* mRNA expression and OS.

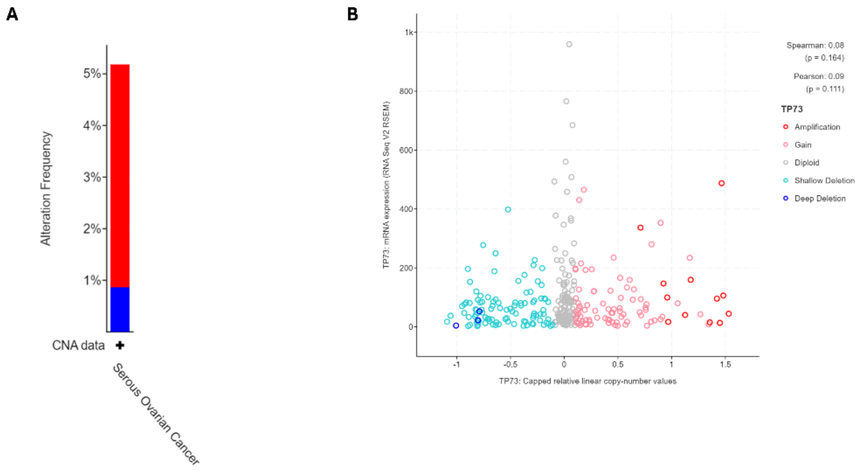

Supplementary Figure S3: Bioinformatics in TCGA cohort. **(A)** The percentage of tumours that have a copy number alteration in the TCGA-OV (Firehose legacy cohort n=579, CNA variation), red shows amplification, blue shows deletion. (**B**) GISTIC plot (capped linear plot) of copy number variation against RNA-seq mRNA levels.

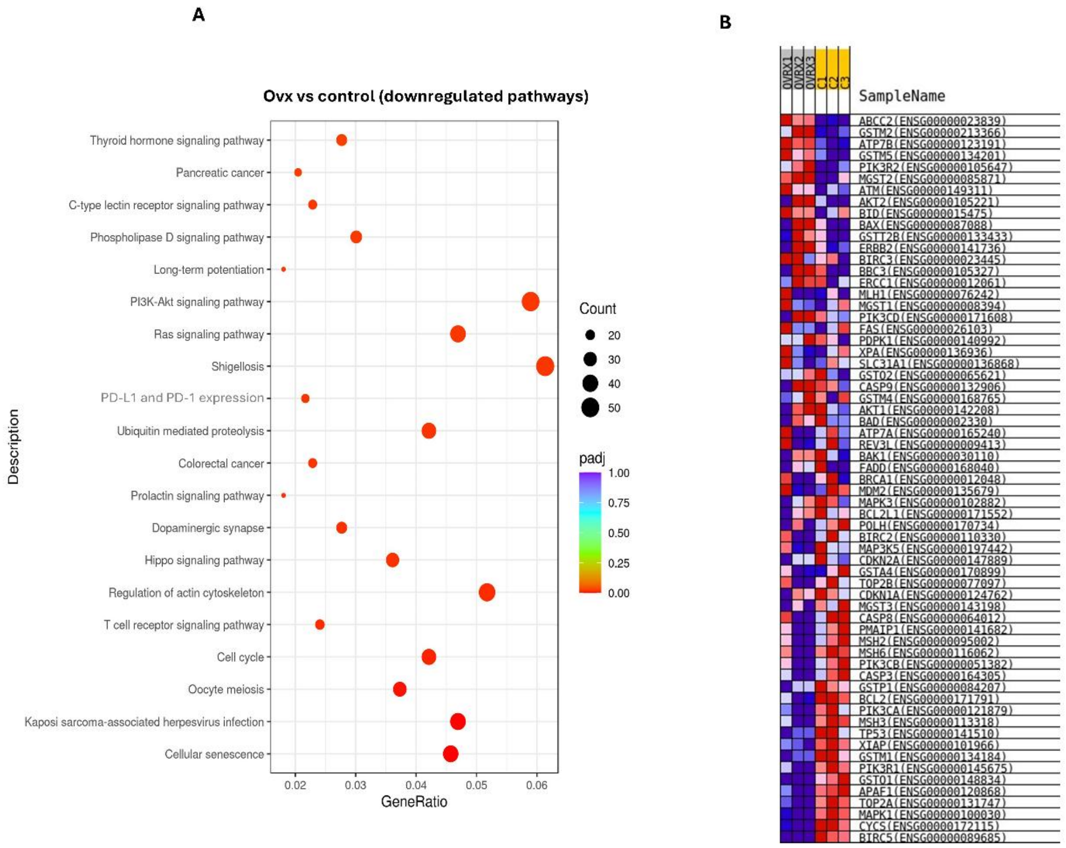

Supplementary Figure S4: Bioinformatics in A2780_p73 ovx cells compared to A2780 control cells. **(A)** KEGG pathway enrichment plot showing significantly down regulated pathways (FDR $p < 0.05$) in A2780_p73 ovx cells compared to A2780 control cells. **(B)** Gene expression profiling of markers involved in platinum resistance in A2780_p73 ovx cells compared to A2780 control cells.

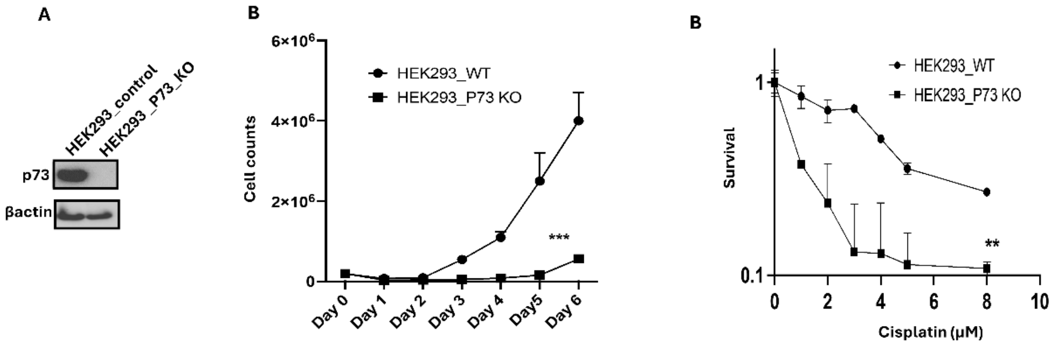

Supplementary Figure S5: (**A**) Western blot showing p73 depletion in HEK293_p73_KO cells compared to HEK293_control cells**. (B).** Cell doubling time assay in in HEK293_p73_KO cells compared to HEK293_control cells. (**C**) Cisplatin sensitivity by clonogenic survival assay in HEK293_p73_KO cells compared to HEK293_control cells.
